# A Software System to Collect Expert Relevance Ratings of Medical Record Items for Specific Clinical Tasks

**DOI:** 10.2196/medinform.3204

**Published:** 2014-02-28

**Authors:** H Benjamin Harvey, Arun Krishnaraj, Tarik K Alkasab

**Affiliations:** ^1^Department of RadiologyMassachusetts General HospitalBoston, MAUnited States; ^2^Harvard Medical SchoolBoston, MAUnited States; ^3^Department of RadiologyUniversity of VirginiaCharlottesville, VAUnited States

**Keywords:** medical informatics, health information management, computerized medical records system

## Abstract

Development of task-specific electronic medical record (EMR) searches and user interfaces has the potential to improve the efficiency and safety of health care while curbing rising costs. The development of such tools must be data-driven and guided by a strong understanding of practitioner information requirements with respect to specific clinical tasks or scenarios. To acquire this important data, this paper describes a model by which expert practitioners are leveraged to identify which components of the medical record are most relevant to a specific clinical task. We also describe the computer system that was created to efficiently implement this model of data gathering. The system extracts medical record data from the EMR of patients matching a given clinical scenario, de-identifies the data, breaks the data up into separate medical record items (eg, radiology reports, operative notes, laboratory results, etc), presents each individual medical record item to experts under the hypothetical of the given clinical scenario, and records the experts’ ratings regarding the relevance of each medical record item to that specific clinical scenario or task. After an iterative process of data collection, these expert relevance ratings can then be pooled and used to design point-of-care EMR searches and user interfaces tailored to the task-specific needs of practitioners.

## Introduction

Adoption of electronic medical records (EMR) has increased dramatically over the past decade, driven in part by sizeable federal subsidies [1,2]. This growth has meant an attendant dramatic increase in the amount and variability of patient data stored in a typical patient’s EMR, creating difficult challenges related to data organization and presentation. As a result, the necessary information to answer a clinical question may be spread among several potentially unstructured documents, requiring a practitioner to undergo a laborious EMR search process. This, in turn, can decrease efficiency, increase medical errors, and generate dissatisfaction among practitioners, potentially negating the safety and efficiency improvements associated with EMR use [3-6]. Difficult-to-navigate EMRs may also contribute to the problem of rising health care costs, because practitioners who are unaware of information contained within the EMR may be more likely to order unnecessary or duplicate tests and procedures [7].

In light of these challenges, the efficiency and accuracy of practitioner data retrieval should be a key focus in the ongoing design of clinical EMR systems and supporting software tools. The addition of advanced EMR search capabilities, such as keyword searches, have improved radiologist efficiency and have the potential to improve patient outcomes [8,9]. To have even greater impacts on clinical care and to improve value, the next generation of EMR technology needs to go beyond keyword searchability and instead present practitioners with a filtered view of the medical record that is germane to their task-specific clinical needs. For example, a radiologist interpreting a magnetic resonance image of a patient’s liver will be interested in a subset of the medical record focused on hepatic and other abdominal issues, along with any history of malignancy. However, a neurologist seeing the same patient for the management of Parkinson’s disease will be interested in a different set of notes, reports, and data. Ideal EMR search algorithms and user interfaces should differentiate between the two practitioners and clinical scenarios. Multiple groups have kick-started this process by developing and validating automated EMR search strategies and data displays for specific clinical tasks, including identification of preprocedural and preoperative risk factors for complications, prediction of long-term mortality of patients admitted to the hospital, and the treatment of intensive care unit patients and neuro-oncology patients [10-14].

A major challenge in designing these task-specific EMR tools for clinical use is obtaining the information about which components of the EMR are most relevant to practitioners in specific clinical scenarios. To overcome this, we propose a strategy for collecting these relevancy data. The proposed approach starts by extracting and de-identifying medical record data from an actual patient in a given clinical scenario. The medical record is then disassembled into component medical record items (eg, radiology reports, operative notes, laboratory results, etc), which are individually presented to a panel of clinical experts. Each medical record item is rated by the experts for its relevance to a specific clinical scenario or task. This process is performed iteratively for multiple patients in the same clinical scenario, thereby creating a robust body of expert-provided relevancy data that indicates which medical record items are most valuable in that particular clinical scenario. The expert-generated relevance data can then be used to design and validate EMR search algorithms and user interfaces tailored to that clinical scenario.

In this study, we describe a software system that we created to implement this process of data collection. We hope this work will serve as the basis for ongoing efforts to improve the value of EMR technology for patients and practitioners.

## Methods

### Tool to Extract Electronic Medical Record Data

A tool was created to extract, de-identify, and format data from our institution’s EMR system according to the defined schema. The first version of this tool was designed around the clinical scenario of a radiologist interpreting an abdominal computed tomography (CT) scan for a patient with a clinical history of abdominal pain. When an index abdominal CT scan matching this specific clinical scenario was identified, the queriable patient inference dossier EMR search/aggregation tool was used to find and extract all radiology reports, operative notes, laboratory results, pathology reports, endoscopy reports, and microbiology results for the given patient within a period extending from 2 years prior to the index radiology exam to 2 years after the index exam [8,9]. These items were selected because they are separately identifiable in our institution’s medical record system and were thought likely to be relevant to common subspecialty clinical situations. A universally unique identifier (UUID) was assigned to the scenario as a whole and for each individual medical record item [15]. The tool automatically removed identifying patient information including the patient’s first and last names, any dates, all physician names, and all identifying numbers (eg, medical record numbers, accession numbers, phone/fax numbers, zip codes, etc). Patient demographic data were reduced to sex and age, with ages greater than or equal to 90 years reported as 89 years to reduce identifiability. Because no look-up table was maintained, re-identification of the patient record was not possible. Although this may reduce opportunities to add additional information to a specific scenario later, it was judged that protecting patient privacy outweighed this loss. The resulting structure was written to an XML file of the format specified in the scenario schema.

### Tool to Collect Rater Scores

**Figure 1:**
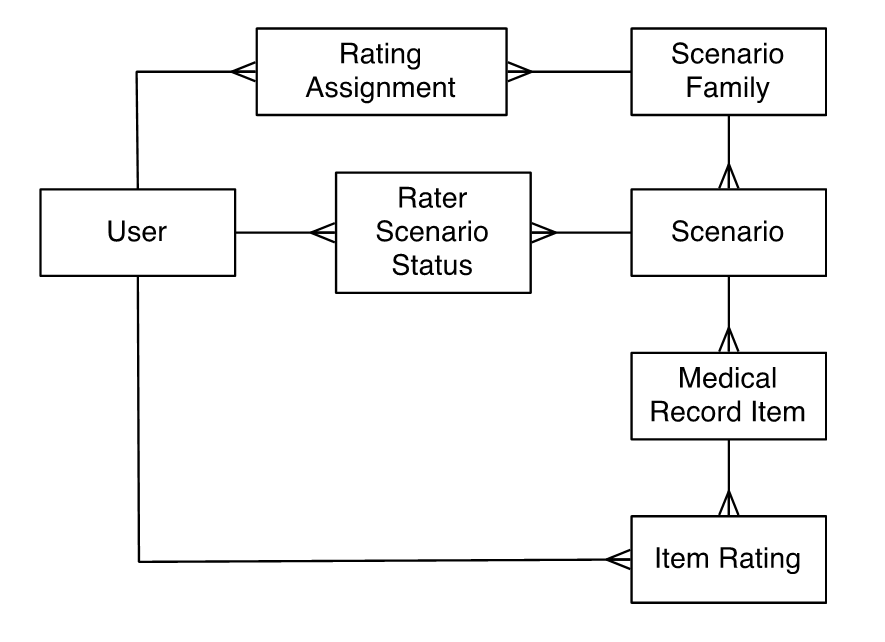
Rating collection tool data model. Inside the collection tool, each set of sample medical records is represented by a ScenarioFamily object, which contains many Scenario objects, which in turn contains many MedicalRecordItems. Expert raters are represented by User objects and are associated with a ScenarioFamily via a RatingAssignment. Raters create an ItemRating object for each MedicalRecordItem within Scenarios belonging to each ScenarioFamily to which they are assigned.

A separate tool was created to manage the collection of expert ratings. The tool was designed to represent incoming sample medical record data sets, information on raters, and the assigned rating scores. The data model to represent these data is presented in Figure 1. This model allows internal representation of incoming medical record data sets as defined in the scenario schema, and exporting of the data into a file according to the scenario family ratings schema. The data model was centered on the ScenarioFamily: data structure; that is, groups of different patients’ de-identified medical records selected and extracted based on a shared clinical context. Each individual case/patient was represented by a Scenario data structure, which in turn is made up of the individual EMR entries for that patient, the MedicalRecordItem objects. When an expert registers to be a rater, a User object was created. Users were then assigned to rate ScenarioFamily objects; this connection was a RatingAssignment. The user’s progress toward completing the Scenarios in the task list of assignments was tracked by RaterScenarioStatus objects. The actual relevance ratings were stored as ItemRating objects. ScenarioFamily objects were assigned UUIDs as needed, as were Users.

Three system interfaces were necessary to permit rater and administrative interaction with the system. The first interface, or task list, showed raters their current set of assignments so they could advance to the next task when finished. The central interface of the system laid out the clinical context for the rater and asked them to assign a relevance score to a medical record item. Finally, an administrative interface was needed for manipulating the scenario families and user assignments and monitoring progress of tasks across the system.

We used open source technologies to implement this system, specifically choosing the Ruby on Rails Web application framework backed by the SQLite3 database engine [16,17]. This resulted in a model-view-controller architecture that could be easily understood and implemented with a variety of platforms and frameworks. The open-source database means that many analysis tools and other software programs can access the generated data as needed. All pages complied with the HTML5 standard to ensure optimal compatibility with modern Web browsers. The Twitter Bootstrap front-end framework and jQuery JavaScript library enabled creation of a richer front end [18]. A Devise authentication plugin was used to manage the process of creating and authenticating raters [19]. We deployed our implementation on a Mac OS X system, but it is possible to deploy the system on most Unix/Linux-based operating systems.

## Results

After obtaining institutional review board approval, the utility of the data extraction tool was demonstrated by extracting data from our institution’s EMR and successfully generating de-identified sample medical record data sets. In general, the automated redaction process was quite effective. However, to ensure maximal protection of potentially sensitive patient information, each data set was further manually examined for residual protected health information, which was then redacted by hand. De-identified data sets were then imported into the database of the rating collection tool using a separate command line utility. The rating collection tool was not actively connected to the EMR system. The scenario data sets were grouped into scenario families; each scenario is composed of a specific example of the clinical context defined by the scenario family.

**Figure 2:**
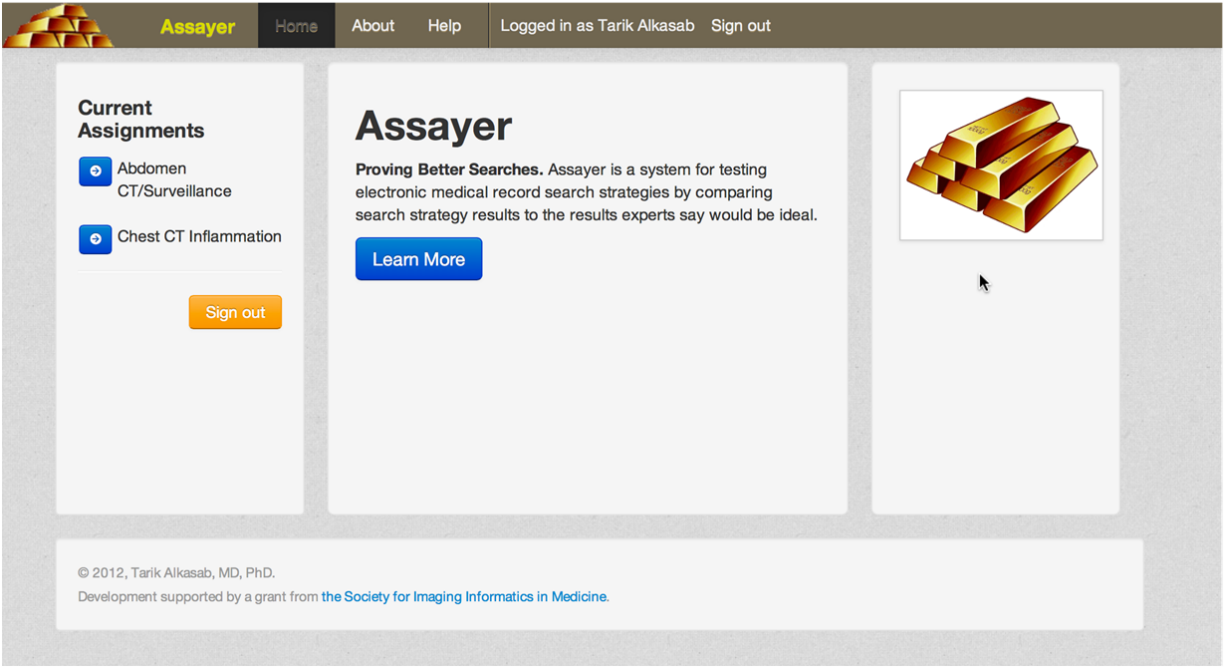
Post log-in page showing assigned tasks. After a user logs into the system, they are presented with a list of their currently assigned clinical scenarios. They can use these links to move directly to the rating interface.

Persons serving as expert reviewers were directed to the system home page, where they could register for a rater account by providing basic personal information to a Web interface. An administrator then assigned each registered user to scenario families based on their clinical expertise using a command line tool. Once scenario assignments were made, the user logged into the system and could see their list of scenario assignments (Figure 2). The rater chose which scenario to work on by clicking the appropriate item from the list.

**Figure 3:**
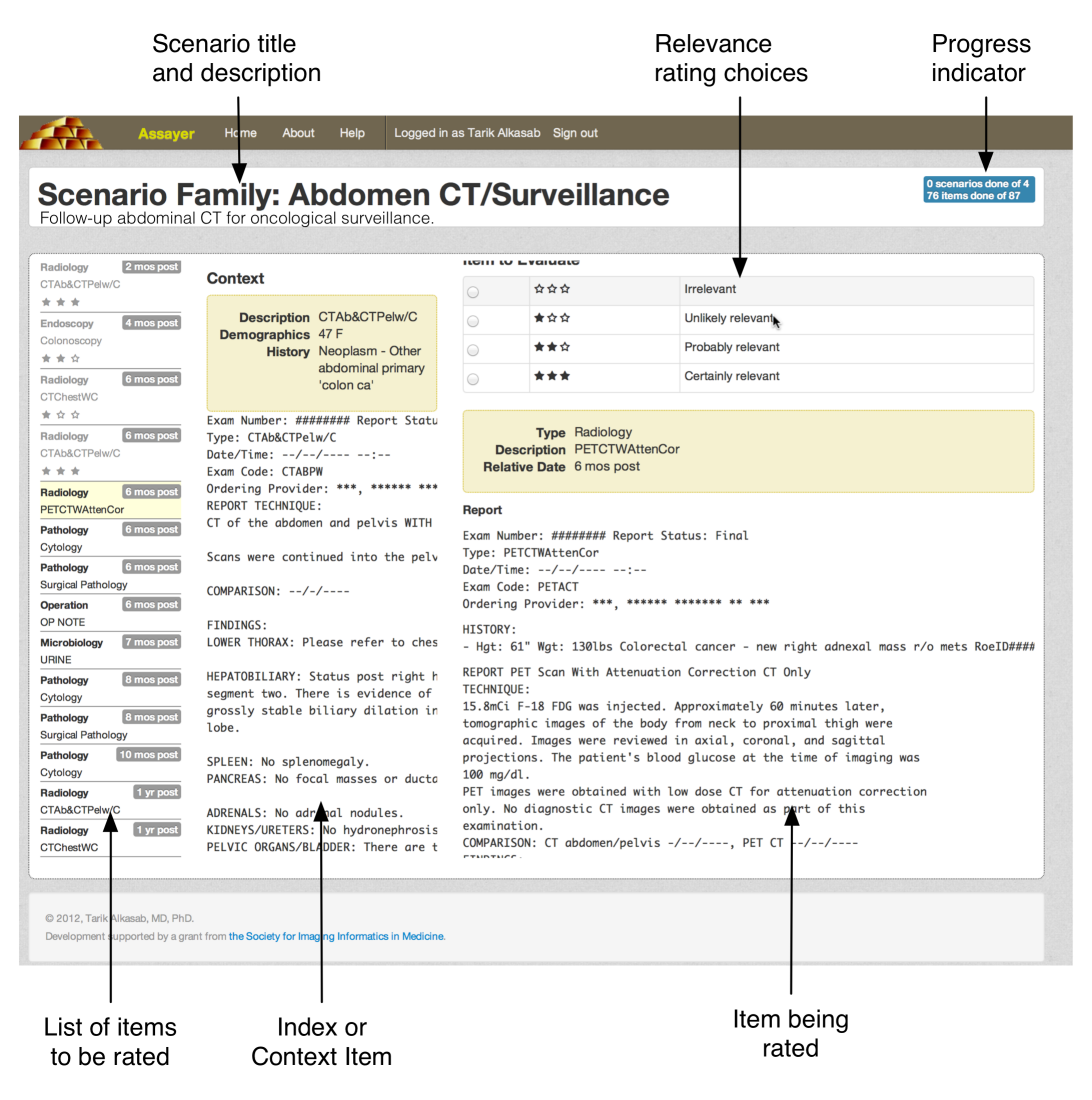
Expert rating interface. An expert rater considers a particular scenario family and a specific clinical context and assesses the relevance of the items in the medical record to the given scenario. Relevance is rated on a 4-step scale: irrelevant, unlikely relevant, probably relevant, and certainly relevant.

After choosing a scenario from the task list, the system brings the user into the main rating interface (Figure 3). The rater is shown the name of the scenario family/clinical context being considered. The central “Context” column shows the specific clinical context of the sample patient whose medical record items the expert user must rate. For example, for the clinical scenario family of a radiologist interpreting abdominal CT scans performed for a clinical history of abdominal pain, the central “Context” column would be the respective clinical history (and possibly the report) of a specific abdominal CT scan that the rater should envision wanting to interpret in that clinical context. Along the left column is the list of medical record items extracted from the EMR of the sample patient in that clinical context. Within the list are both the medical record items that still need to be rated, along with the items that have already been assigned a relevance score by the rater. The rater can scroll up and down this list to review the items that they have already rated and the relevance scores assigned to those items. An indicator of the rater’s progress through a given clinical scenario (ie, an individual patient) and the scenario family more broadly (ie, the assigned cohort of the sample patients) is shown in the upper right portion of the screen.

When the rater clicks on a specific medical record item in the list on the left-hand column of the screen, the system presents the rater with the medical record item in full detail in the right-hand column along with the choices for rating relevance. The rating relevancy choices are represented both as words (“irrelevant,” “unlikely relevant,” “probably relevant,” and “certainly relevant”) and as a number of filled-in stars (0-3). Once the rater clicks on a relevancy rating, the system automatically presents the next medical record item in the right-hand column. When all of the medical record items have been rated in a given scenario, the rater is taken to the first item in the next scenario in the scenario family. Likewise, when all of the scenarios in the scenario family have been rated, the rater is returned to the home page, where they can choose to proceed to the next uncompleted assignment.

**Figure 4:**
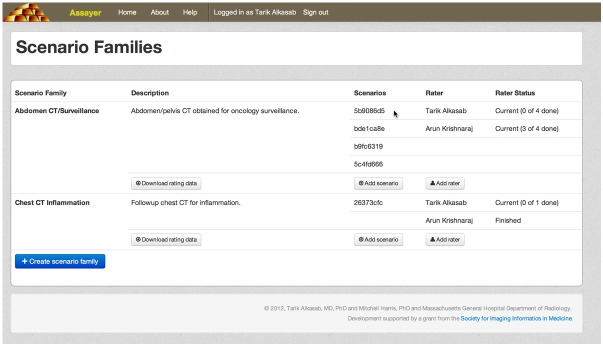
Administrator monitoring interface. An administrator keeping track of activity on the service can view the monitoring page, which shows all of the active scenario families, the scenarios making them up, the raters assigned to evaluate the items, and their progress in rating all of the items. Tools for defining new scenario families, uploading scenarios, and adding raters are also available from this page.

An administrative user can track the progress of raters through their assigned scenario families via an overview interface (Figure 4). This interface displays a list of the scenario families known to the system and abbreviated UUIDs for its component scenarios. The overview interface displays the current progress of each rater in their task list, for all raters assigned to a particular scenario family. For each family, links are provided to add a new rater, upload a new scenario, or download the current ratings data.

Finally, once the assigned panel of raters has rated the relevant items for the individual scenarios in a scenario family, an XML file containing the relevant rating data can be extracted. These ratings data can then be used to design and validate tailored medical record search strategies and user interfaces for a given clinical task.

## Discussion

### Principal Results

We have successfully designed and implemented a system for extracting exemplar medical record items, such as laboratory results and operative reports, from the EMR and obtaining task-specific expert relevance ratings for those items. The extraction tool pulls a subset of the medical records for a patient, de-identifies it, and formats it so that it can be included in the rating collection tool. The rating collection tool then allows a clinical expert, such as a radiologist for the clinical application described herein, to review and rate the component parts of even complex medical records, thereby highlighting the items in the medical record that are most relevant to a specific clinical task. Both the de-identified clinical information and the expert-supplied relevance ratings are captured, organized, and exported in a format that can be used for search strategy optimization and the design of tailored EMR user interfaces.

The design of this system was based on a few guiding principles. First, the system should be built using easily available, well-understood open source tools according to standard design patterns. Second, the system should easily fit into a broader framework for designing medical record searches and user interfaces, including, but not limited to, importing and exporting open formats to simplify data interchange. Finally, the system’s interface with expert raters should be simple and efficient.

Based on these principles, we created a system to reduce the effort associated with the collection of expert ratings data, while ensuring the accuracy and robustness of the data collected. Recognizing the high value of an expert’s time, efficiency of the process was an absolute requirement. Thus, whenever an expert begins a session, he or she is moved into the process of examining and rating medical record items as rapidly and efficiently as possible. The tool also allows multiple experts to evaluate the same body of de-identified patient data. Having multiple raters review the same medical record items reduces the effect of individual rater idiosyncrasies. Moreover, new scenarios can easily be added to a family to reduce the effect of specific variations within a single source medical record, if needed. Last of all, the system allowed experts to be matched with clinical scenarios specific to their expertise.

### Limitations

With the software system implemented, the most important challenges to actually putting the system into use revolve around selecting an appropriate clinical scenario and recruiting appropriate experts. With regard to clinical scenarios, it is important to make the scenario specific enough so that the expert raters think they are making a concrete decision about relevance rather than an abstract one. Expert recruitment, on the other hand, benefits from selecting both highly specialized experts and more generalist practitioners. To maintain the raters’ interest, everything possible should be done to reduce administrative overhead on the raters.

### Conclusions

Looking forward, it will be important to develop a capacity to facilitate interchange between different sites or installations to maximize the generalizability of the collected ratings data. Even though the medical record items are de-identified, such interinstitutional collaborations would likely require access to be restricted to a predefined group rather than the world at large due to the potentially sensitive nature of the information. This could be accomplished by allowing each site to maintain a list of partner sites where a combination of sample medical record items and ratings data could be securely exchanged. Once established, exporting scenario data, scenario family data, and ratings data could then be available via a website. In this way, a broader range of experts and sample clinical items could be assessed, leading to a more robust body of expert ratings data.

We envision a multicenter project to collect expert relevance ratings for several clinical scenarios common to radiology. In this project, sample medical record items will be pulled from each center and pooled into a common sample set for each clinical scenario. The pooled expert relevancy ratings data can then be used to validate candidate search strategies and eventually to develop filtered views of the medical record specific for clinical tasks commonly faced by radiologists. Obviously, such interinstitution synergy poses many challenges, not least of which is the incompatibility of medical record data formats. As institutions begin such collaborations, it is important to define standards for representation of the medical record information. This could even be expanded to account for international differences. It will also likely be necessary to expand the data model to clearly state which experts should be allowed to view data from which partner institutions. These additional efforts would be rewarded by a much richer set of sample medical record data and ratings.

The approach we outlined emphasizes a “wisdom of crowds” data-driven approach to identifying likely-to-be relevant medical record information rather than an expert-driven methodology. We designed the tool described herein to gather this collective intelligence because it is much harder to incorporate such information into a design process. In fact, we believe that the most effective search strategies will be generated by starting with hyper-local experts, who define relevance based on their specialized experience, and then proving and testing their designs against crowd-sourced data. Far from denigrating the potential contribution of individual innovation, we hope to provide a way to hone those contributions. Future versions of the software could allow the raters to provide specific comments and notes to search designers to further spur these efforts.

In sum, context-specific EMR searches and user interfaces have the potential to increase the efficiency and safety and reduce the cost of health care delivery. To achieve these ends, development of these tools must be data-driven and influenced by an understanding of practitioner information requirements. The data collected using the herein described software tool can serve as the basis for acquiring this essential guidance, with the ultimate goal of creating tools that allow physicians to rapidly and effectively navigate EMR systems. By providing this as an open-source tool with open formats for data interchange, we hope to bolster the adoption of the process through interinstitutional synergy.

## Abbreviations

CT: computed tomography
EMR: electronic medical record
UUID: universally unique identifier
